# Emergency online school learning during COVID-19 lockdown: A qualitative study of adolescents’ experiences in Italy

**DOI:** 10.1007/s12144-021-02674-8

**Published:** 2022-01-07

**Authors:** Iana Tzankova, Christian Compare, Daniela Marzana, Antonella Guarino, Immacolata Di Napoli, Alessia Rochira, Emanuela Calandri, Irene Barbieri, Fortuna Procentese, Flora Gatti, Elena Marta, Angela Fedi, Giovanni Aresi, Cinzia Albanesi

**Affiliations:** 1 Department of Psychology, University of Bologna, Piazza Aldo Moro 90, 47521 Cesena, Italy; 2 Psychology Department, Università Cattolica del Sacro Cuore, Largo Gemelli 1, 20123 Milan, Italy; 3 CERISVICO Research Centre on Community Development and Organisational Quality of Life, Via della Garzetta, 50, 25133 Brescia, Italy; 4 Department of Humanities, University of Naples Federico II, via Porta di Massa 1, 80133 Naples, Italy; 5 Department of History, Society and Human Studies, Laboratory of Applied Psychology, University of Salento, Via di Valesio, 73100 Lecce, Italy; 6 Department of Psychology, University of Torino, via Verdi, 10, 10124 Torino, Italy

**Keywords:** online learning, distance education, emergency schooling, adolescents, pandemic, COVID-19

## Abstract

The COVID-19 pandemic caused abrupt and profound changes to teaching and learning. The present study seeks to understand adolescents’ experiences of the emergency adoption of online school learning (OSL) during the first national lockdown in Italy. Sixty-four students in their final two years of high school were interviewed and content analysis was performed. The findings describe students’ views of the changes related to OSL according to structural, individual and relational dimensions. Schools’ lack of organization, overwhelming demands, as well as experience of difficulties in concentration, stress and inhibited relationships with teachers and classmates were among the challenges evidenced in the transition. OSL, however, has also made it possible to experience a new flexibility and autonomy in the organization of learning. The study stresses the importance of fostering adaptation of teacher-student relationships and collaborative learning in order to improve schools’ preparedness for digital transitions in and out of emergencies.

In the immediate aftermath of the COVID-19 outbreak, the Italian Government, like many others, endorsed the suspension of in-presence school lessons. The Italian Ministry of Education decided to implement an online version of schooling – online school learning (OSL) to flatten the infection curve. This reactive response to the pandemic disrupted traditional teaching practices and the daily routines of thousands of students and their families. Education systems worldwide had to face an unprecedented challenge, trying to deliver education remotely through a mix of technologies to ensure continuity of learning for all.

In some cases, OSL tends to change the face of education toward a more effective and enjoyable experience (Lestari & Gunawan, 2020). However, OSL can be complicated and may not be equally suitable for all students (Cavanaugh et al., 2009). The stress and isolation related to the pandemic and the uncertainty surrounding the emergency-based adoption of OSL can further negatively impact the learning experience (e.g., Sutarto et al., 2020) and adolescent development and well-being (O’Sullivan et al., 2020) – especially in contexts where school preparedness is low and students lack confidence in technology use. The development of adequate responses by the educational system to critical situations such as lockdowns is of paramount importance to promote more effective and beneficial learning and students’ resilience. Listening to students’ perspectives on OSL can inform future distance education strategies and help schools and teachers develop classroom activities. The present study aims to understand adolescent students’ experiences of the early adoption of online school learning methods during the first national lockdown in Italy. We explore upper secondary school students' perspectives on the changes produced by the abrupt emergency shift to OSL concerning teaching, learning, and relationships with peers and teachers.

## Online School Learning and the COVID-19 Emergency in Italy

The existing literature has investigated technology-mediated learning by using various terms and focusing on specific applications – e.g., distance learning, online learning, remote learning, e-learning, blended learning, tele-learning, open learning, etc. Generally, distance education can be defined as “teaching and planned learning in which the teaching normally occurs in a different place from learning, requiring communication through technologies, as well as special institutional organizations” (Moore & Kearsley, 2012, p. 2). In line with this definition, throughout this paper, we mean with *online school learning* the teaching and learning that relies on communications through online technologies in the context of primary and secondary schooling institutions.

In a pre-COVID world, distance-based methods for education had already received growing attention since the ‘90s as digital technologies allowed for their increasing implementation. The field of research before the COVID-19 pandemic had focused mainly on higher education and lifelong learning, although literature on distance education at primary and secondary school levels had also been growing (Barbour, 2019). For example, Barbour and Reeves (2009) reviewed the literature on the growth of virtual schooling at the K-12 level in the US. In Italy, experimentation and application of digital technologies in schools had also grown in the last decades, albeit slowly and with resistance to the modernization of educational spaces and methods (Dominici, 2015). Distance education in primary and secondary Italian schools has been rare and mostly related to specific social inclusion needs (Roncaglia et al., 2021).

Overall, the Italian school system is regulated at the National level through common educational standards, but schools also have significant autonomy. The system is mainly public and is regulated by an inclusive principle. Bombardelli and Codato (2017) stressed that the Italian school system relies heavily on traditional teaching methods – face-to-face lessons and individual study and assessment with limited group activities. The PISA Report 2018 (Organization for Economic Co-operation and Development [OECD], 2020) showed that students in Italy believe that few teachers have the necessary technical and pedagogical skills to integrate digital devices in instruction. Among Italian teachers, 35.9% also reported a high need to develop their ICT skills for teaching. Moreover, students were one of the less cooperative compared to other PISA-participating countries. In this context, the COVID-19 emergency could exacerbate some of the pre-existing issues in schools’ and teachers’ preparedness for online school learning.

Indeed, the forced transition to OSL has presented further problems in applying effective teaching and learning strategies while putting significant pressure on families (Roncaglia et al., 2021). In this sense, it has been highlighted that the adoption of online learning methods in response to a crisis, as in the COVID-19 pandemic, should be considered a distinct and temporary type of instruction due to the lack of planned design and implementation (Hodges et al., 2020; Williamson et al., 2020). However, this forced experience can inform future preparedness of educational institutions to address other potential emergency disruptions and to meet students’ needs for quality learning and for interactions that can improve their outcomes, health, and well-being (O’Sullivan et al., 2020). Thus, it is essential to shed light on the learners’ experiences of OSL during the COVID-19 crisis and the perceived characteristics that can influence them.

## Characteristics of Online School Learning

At the primary and secondary levels, OSL can differ on a number of aspects that are relevant to the potential benefits or challenges stemming from its adoption, including comprehensiveness, type, location, delivery, type of instruction, teacher-student interaction, and student-student interaction (Barbour, 2019). We adopt an ecological perspective, from community psychology, to analyze and understand the complexity of students’ experience of these aspects in OSL within multiple levels of the school ecological environment (Trickett & Rowe, 2012).

Based on the characteristics of OSL that have already been studied in the literature, and in accordance with an ecological approach distinguishing between different levels of analysis in the process of adaptation to OSL during the COVID-19 emergency, we identified different factors that can condition the perception of OSL among students at the structural and organizational, personal and psychological, and interpersonal and community levels. At a structural and organizational level, OSL can be organized differently according to technology, types of programs and delivery, pedagogy, monitoring activities, and role of assessments. At a personal and psychological level, students’ differing needs and resources can determine variations in the learning experiences in OSL. Finally, at an interpersonal and community level, the quality and nature of teacher-student and student-student interactions may vary, making the sense of a learning community crucial. Below, we review the main relevant aspects that can shape students’ perceptions of OSL during the COVID-19 pandemic in light of the literature on the topic.

### Structural and Organizational Factors

In general, OSL has been promoted as offering numerous advantages for schools: providing greater access to education, meeting special needs of students, offering courses otherwise unavailable at the school (e.g., in rural settings), reducing scheduling conflicts, etc. (cf., Barbour & Reeves, 2009). Flexibility is a particularly attractive feature of OSL as it can allow students to manage their learning time and location according to their specific needs.

However, existing inequalities related to Internet access and individuals’ familiarity and engagement with technology can determine different levels of readiness for OSL (Dray et al., 2011), which could undermine the democratic potential of distance education. The COVID-19 crisis seems to have deepened existing inequalities among students, as the digital gap and students’ dropout has increased (Hall et al., 2020). Students from more disadvantaged groups are reported to have less access to remote learning technologies and are less likely to do schoolwork during the day (Asanov et al., 2021). The pandemic unveiled that most students attended school at distance in challenging circumstances and often without authentic teaching or support from their teachers (UNESCO, 2020). The Italian National Institute of Statistics (ISTAT; www.istat.it) reported that 45% of youths (age 6 to 17) struggled to cope with OSL due to a lack of devices in their homes. Moreover, the COVID-19 lockdown conditions resulted in a shift in family life and schooling, in which students relied heavily on their families. Parents and caregivers would be involved in providing access to internet, technology, and adequate workspace; in some cases, they were also primarily responsible for their children’s homeschooling (Thorell et al., 2021).

The levels of teachers’ supervision and online management are also relevant factors in the online engagement of high school students (e.g., Hawkins et al., 2013). The pandemic, however, seems to have amplified possible differences in teachers’ skills and readiness to teach online. In some cases, teachers became more innovative and creative in developing methods to engage students, while in other circumstances they reported several difficulties (Lestari & Gunawan, 2020; Trust & Whalen, 2020). Teachers’ preparedness and previous experience with online teaching were crucial factors in managing the complexity of technical, social, and pedagogical challenges, government guidelines, and students’ needs (e.g., Sokal et al., 2020).

### Personal and Psychological Factors

Other challenges associated with virtual schooling include personal differences in student readiness, learning styles, and motivation. In particular, students who are more successful in OSL environments tend to prefer independent learning, have higher intrinsic motivation, and have greater time management, literacy, and technology skills (Cavanaugh et al., 2009).

OSL programs should therefore be designed to maximize potential benefits and support students’ motivation. In the emergency transition to OSL, pedagogies supported by an effective employment of technology (e.g., using different platforms as repositories for learning materials, adapting activities to students’ conditions in their homes and neighborhoods, facilitating online small-group activities, providing asynchronous use of technology and multimedia resources) facilitated motivation, collaboration, and authentic learning activities, enhancing students’ learning in the pandemic situation and could be applied during future emergency events (Yates et al., 2020).

In general, student satisfaction is strongly related to a more favorable use of digital devices when teachers show technological ability and willingness for using them (Valantinaitė & Sederevičiūtė-Pačiauskienė, 2020). Indeed, students can develop their virtual competence, which is positively related to course satisfaction (Wan et al., 2008).

Distance education studies have also emphasized the importance and the challenges of developing autonomy in the OSL process. Lewis et al. (2014) found that students need adequate learning strategies such as orientation to the learning environment and guidance to increase OSL readiness. Students need to feel supported and welcomed in the OSL environment, and technology should not be a barrier to successful learning. Research shows that autonomy support within the learning context leads to more self-determined motivation among learners (Hartnett, 2016).

### Interpersonal and Community Factors

According to Rovai (2002), virtual classrooms can develop a sense of community comparably to traditional teaching by promoting belonging, trust, interaction, and commonality of learning goals. Indeed, Ke et al. (2011) showed in a qualitative study that supporting interaction online requires more collaborative processes, including peers’ discussions about course subjects, sharing and generating ideas, and using them for problem-solving.

Recent studies highlighted the benefits of a supportive educational community in facilitating the online transition for students and teachers who felt less isolated and less stressed (Procentese et al., 2020). Peterson et al. (2020) reported that the schools that prioritized relationships and students’ well-being with practical strategies cope better with OSL.

However, maintaining relationships was the most significant challenge teachers, students, and schools faced in emergency OSL – the lack of togetherness with peers seems to be one of the most critical factors (Sutarto et al., 2020). Some authors reported that secondary school students missed traditional courses because they found them more motivating, fun, understandable, and socially meaningful (Niemi & Kousa, 2020; Pınar & Dönel Akgül, 2020). Indeed, schools have multiple functions in students’ lives, among which are establishing social relationships (keeping company with peers, chatting, playing, having lunch and coffee together, etc.) and learning to collaborate (working together, sharing ideas, and mutually support learning; Wentzel & Watkins, 2002). Despite these functions having a substantial impact on students’ well-being and learning motivation, students reported that these elements were not present enough in OSL during the pandemic (Black et al., 2021; Niemi & Kousa, 2020).

## The Present Study

The main research question for the study was: how did students experience changes in schooling due to the adoption of emergency OSL during the initial phases of the COVID-19 crisis in Italy? Therefore, the aim was to explore and analyze adolescent students’ perceptions regarding OSL, with particular attention to the relational processes (among students and between students and teachers) and the teaching and learning changes produced by OSL. It focused on the unexpected and abrupt shift to OSL, which was needed to continue educational activities under lockdown measures. More specifically, an explorative qualitative approach was adopted to identify benefits and limitations in the structural/organizational, personal/psychological, and interpersonal/community characteristics of emergency OSL according to students’ perspective.

## Method

### Participants

The study involved as participants 64 adolescents, of whom 35 were female (54.7%). Participants were recruited via snowball sampling in Northern and Southern regions in Italy by research units from five universities: University of Bologna, Università Cattolica del Sacro Cuore, University of Naples, University of Salento, University of Torino. The criteria for inclusion in the study required participants to be enrolled in the last two academic years of high school. Participants from local schools and associations were approached at the end of the National COVID-19 lockdown. Those who agreed to be interviewed were asked for contacts of friends, schoolmates, and peers from associations. Snowballing continued until the sample included gender balance, different school tracks, and a diversity of experiences of extra-school activities. Age ranged between 16 and 19 years (*M*_*age*_=17.72; *SD*=.65). Fifty participants (78.1%) attended lyceum (e.g., humanities, scientific, art), and 21.9% vocational-technical high school. Almost half (48.4%) were part of associations (i.e., sport, religious, scout). The majority of participants lived with parents and one or more siblings (73.4%), 4.7% with parents only, and 21.9% in different family compositions (e.g., grandparents, mother or father, parents and grandparents). The majority of participants (67.2%) lived in towns, 4.7% in cities, and 28.1% in metropolitan areas in Italy's northern (54.7%) and southern (45.3%) regions. The diversity of migrant or ethnic backgrounds was not taken into account as a criterion for inclusion in the study. Moreover, information about the social and economic background of the participants and their families was not asked during the interviews.

### Study Design

Ethical clearance for the research was obtained by the Ethics Committee of the University of Naples. Before the interview, participants were asked to read and sign an informed consent form describing the study. For underage participants, informed consent was also signed by their parents or caregivers.

The data were collected through one-to-one semi-structured interviews conducted between May and June 2020, following a guide designed to collect participants’ perspectives on the COVID-19 pandemic, consequent restrictions, and their influence on everyday life, social relationships, and community impact. The interviewers were research assistants who were trained in interviewing methods and were of age as close as possible to that of the participants, in order to facilitate the relational quality of the interactions between interviewer and respondents and reduce misrepresentations due to social tuning (i.e., the tendency to adjust beliefs and utterances to match the interlocutor; Sinclair et al., 2005). Participants were asked to elaborate on: their experience of emergency restriction measures (e.g., “How are you experiencing this period of restrictions linked to COVID-19 emergency? Which of the changes taking place is weighing the most on you? Is there any aspect of this situation you are more comfortable with?”); how they reorganized their daily routines (e.g., “How did you reorganize your day?”); the changes they saw in their relationships with family members and peers, in school learning, in extra-scholastic and associational activities (e.g., “How have your family relationships changed? And what about the relations with your friends? How has teaching at school changed? What do you like and what do you dislike about this way of doing school?”). Additionally, they were invited to expand their view of their impact on local, national, and global communities in the pandemic context. Questions were open-ended to ensure the spontaneous emergence of topics, while follow-up questions were posed as needed. This paper focuses specifically on interview findings related to perceptions of online school learning and the related changes in the school environment and relationships.

The interviews were audio-recorded and transcribed verbatim. The organization and analysis of the data were aided by the software Atlas.ti.

### Analysis

We adopted a directed approach to content analysis (Hsieh & Shannon, 2005). In this approach, researchers identify key concepts to define the initial coding categories following previous research and findings (Potter & Levine-Donnerstein, 1999). Since the initial coding follows an assignment of predefined codes according to the existing literature, this approach can be defined as *deductive category application* (Mayring, 2015). A mixed procedure complemented the analysis, whereby the deductive category system was further refined and supplemented with new codes in a process of *inductive category formation* (Mayring, 2015).

According to the literature above and the research aims in the present study, the main online school learning and relational domain codes were identified a priori. The first reading of a part of the interviews (about 20%) was carried out independently by coders within each research unit. This first reading was aimed at assessing the category system and verifying the existence and relevance of a priori themes within the corpus. For each category, other codes were outlined, allowing the designation of further aspects of the predefined categories. The coding system was discussed within the overall research team in order to reach an agreement about the appropriate structure and meaning of codes. Any disagreement in coding was carefully scrutinized and reconciled through discussion and consensus among all research members. Special attention was paid to quotations that did not fit in the developing coding system (negative case analysis) and the categories and codes were reassessed if needed. The resulting a priori and new codes were listed in a shared codebook, uploaded on an online folder accessible by all researchers. The entire corpus of the interviews was consequently encoded with the a priori and new codes.

## Results

**Fig. 1:**
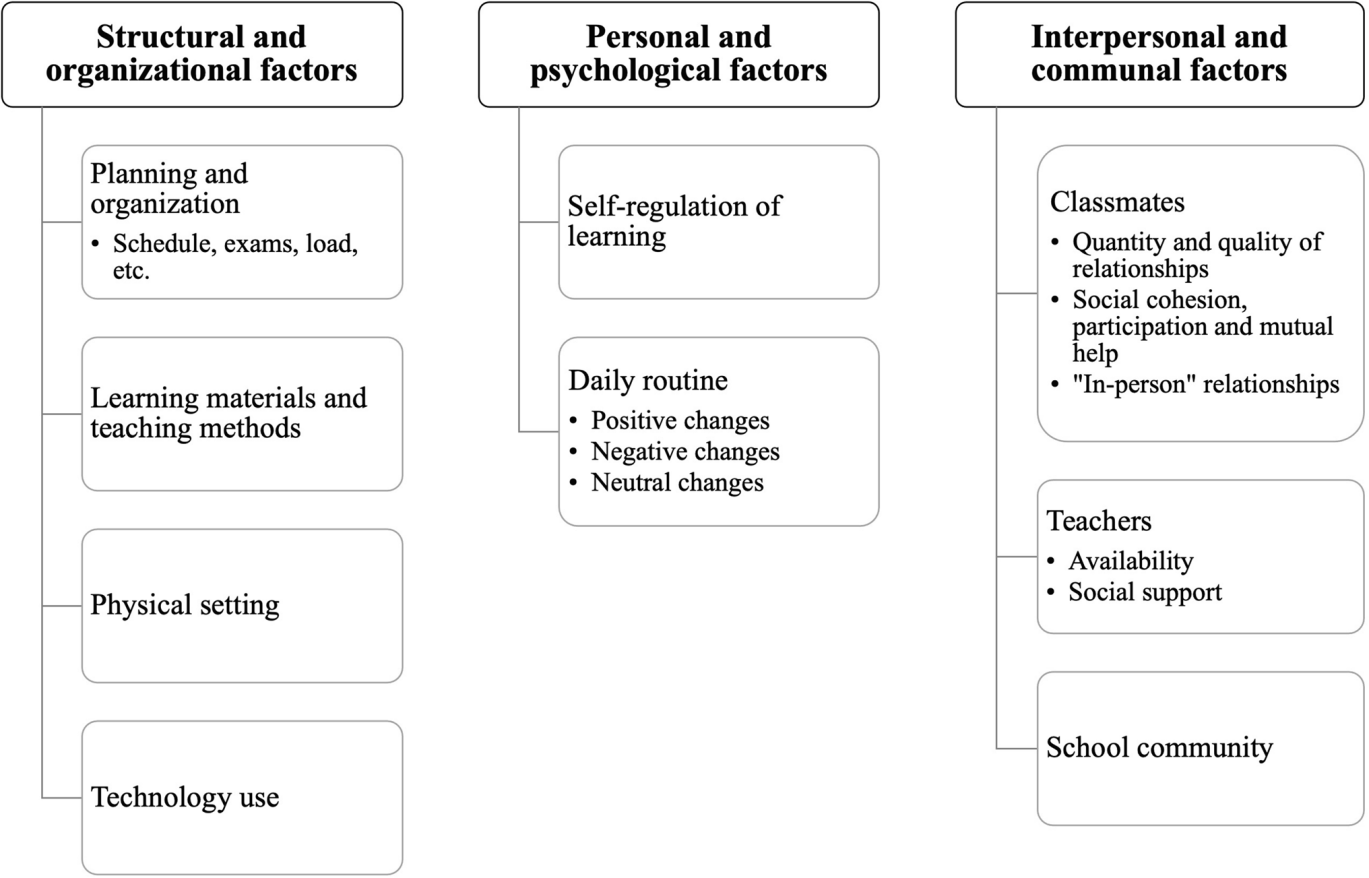
Results: Macro-categories and themes

The results were grouped into three macro-categories of changes, in line with the study’s objectives: 1) structural and organizational factors of OSL; 2) personal psychological factors of OSL; and 3) interpersonal and communal factors of OSL. Figure 1 shows the macro-categories and the main themes identified within them. The first macro-category refers to changes related to the organization of OSL in terms of school planning, learning materials and methods, physical setting, and technology use. The second one collects themes about self-regulation for the OSL modality and the reorganization of one's daily routine linked to the OSL. Finally, in the last macro-category, there are the themes related to the change that this modality has had in school relationships with classmates, teachers, and school as a community. In the following sections we used “verbatim” quotation to elucidate participants’ perspectives and the main themes that emerged across interviews with the intent to show their variety (Eldh et al., 2020).

### Structural and Organizational Factors of Online School Learning

#### Planning and Organization

Students highlighted that the emergency and the abrupt changes had brought disorganization in their schools, revealing unpreparedness to adopt OSL and to adjust methods and study load. An overall sense of inaction and passivity seems to be the interpretation frame offered by participants. Sometimes students were positively surprised by the school’s capability to adapt to the emergency. However, students often perceived school as incapable of coping with the emergency, passively trying to shift in-presence activities and organization to the online setting. They reported being frequently confused when it came to the planning of examinations, schedules or staying connected and interacting with their teachers:

It’s challenging to follow everything that happens. It’s not clear when or what tests will be about, and it’s difficult to ask because you don't see all the professors every day, so you must always ask your classmates.

Participants also highlighted that, requests related to their courses could grow excessively over time, and the schedule could be too demanding. It was reported that there were teachers who would feel justified to prolong lectures and require studying more content than usual, suggesting a sense of lack of respect for students’ needs for personal time: “Some professors manage time and workload well, some very poorly, making their lessons longer and giving us too much homework”.

#### Learning Materials and Teaching Methods

When teachers were unable to adapt their teaching to the situation, overloading students with assignments and tests, proposing the same format of “off-line teaching”, students considered their work ineffective and dull, as well as unsupportive:

Some teachers had been difficult to follow even in the classroom, and to see them in camera getting lost; I admit that I’ve turned everything off and listened, but I struggled to follow because it was impossible to understand.

At the same time, interviewees perceived OSL as more satisfying than classroom teaching, when there was greater learning flexibility (e.g., asynchronous materials) and creative teaching methods, more planned examinations, and reduced pressure. These changes could facilitate some students, increase their motivation to follow lessons, and promote self-regulation of learning. Learning materials could help in-depth studying. Indeed, students appreciated the asynchronous learning and quick accessibility of learning materials. For example, students appreciated teachers who used online tools creatively in providing contents and explanations: “I have a teacher who sends us audios on WhatsApp besides classes. Taking notes with these audios was useful. If you don't understand something, you can go back and listen again”.

#### Physical Setting

Another aspect that characterized the experience of OSL for students was the change of setting. The opportunity to be at home could provide comfort and facilitate time management and autonomy for many students. However, there could also be too many distractions in this setting:

This is not school [ …] it was hard to stay focused. Notifications pop out on the smartphone, and you get distracted.

Students considered the availability of computers and the Internet in teaching as “the right opportunity at the right time", considering that, without technology devices, schooling in the COVID-19 emergency would not be possible: “The online school was the only way to get the programs and the lessons going, and this is something we are lucky for”.

Moreover, OSL had brought greater awareness that technology can be used in multiple ways and can be part of learning, not just fun:

I discovered new ways to use the phone. Besides Instagram or social media, I discovered the utility of the phone itself, which is much more useful, even for doing homework. I found a new and helpful way to use it.

### Personal and Psychological Factors of Online School Learning

#### Self-Regulation of Learning

OSL could allow students to organize their studying activities, self-regulate their learning, and acquire more autonomy in the school context. This could also leave space to pursue personal interests within the taught subjects. This self-organization was associated, for example, with how students expected that more advanced studies at the university level would be:

I liked the slower pace and the fact that I can organize the study independently. It's like an exercise for the University, where there won't be teachers who follow you day by day, but you have to self-organize your study.

Students also appreciated when they were given assignments to complete independently as this facilitated concentration:

I feel independent work to be effective; instead of spending an hour and a half listening to teachers speaking, perhaps not following them, they gave us assignments that will be evaluated. The commitment and the attention or concentration I put into it are different*.*

The possibility of self-regulation in learning was found to be stimulating and beneficial. OSL could also be more suitable to students’ characteristics, help their anxieties and even improve their learning outcomes: “I had positive results, and it goes much better now than it used to. Maybe because I feel more comfortable in front of the pc screen since I am timid and very anxious”.

However, the experience of a new way of online-based evaluation could influence emotional states negatively:

The idea of standing in front of a computer alone or even having tests scared me a little more because in class, even if something is wrong, there are always people close to you, while the idea of having a test alone in my room made me a little more afraid.

#### Daily Routine

Adolescents’ daily routine is undoubtedly an aspect that has suffered the impact of the lockdown. OSL and the inability to go out and spend time with people outside the home have changed adolescents’ daily organization. These changes could be substantial or just minor adjustments. Moreover, they could be evaluated positively, negatively, or with no specific meaning.

##### **Positive Changes**

Forced closure at home and changing daily routines was an opportunity to engage in hobbies, learn new things, or pursue interests that had previously been set aside or undervalued:

I find myself closed at home, it’s true, but I have time to do all the things I couldn’t do before. I’m still going to school, and I’m doing more for myself. I never had time for reading, and now I have it*.*

Students’ daily routine seems to have changed because school life had changed. The transition to schooling at home was felt particularly in its impact on students’ transport use, sleeping routine, use of house spaces for learning, etc. The new daily organization also made it possible to have more time for rest and sleep, conditions that adolescents appreciated:

I realized that life was much faster before the quarantine, and I noticed it not much from my daily routines but my physical and mental state, from my tiredness. And for meals, lunch is earlier, and I have much more time*.*

##### **Negative Changes**

The change in daily routine also represented a difficulty. The condition of loneliness at home and lack of socialization with peers led to experiencing fatigue in everyday life. The precariousness of the situation and its persistence over time has brought a crisis in the task management for adolescents who often, in the absence of a good organization by the school, had greater difficulties adapting. The change in the pace of work and organization in learning represented the area of greatest difficulty. Students could perceive school as a “never-ending task”: “It seems that school has become more demanding. I work ten times more than usual, so it's a little bit more difficult”.

Above all, participants evidence a “fatigue” effect stemming from the prolonged condition of isolation, increased focus on studying, and disproportionate technology use. While they found positive aspects in the new situation at the beginning of the lockdown, over time, monotony took over. Moreover, the amount of screen time during the day could be excessive and independent learning could be elicited not so much by the organization of OSL but by its inefficacy:

You realize that every day you wake up, you put yourself in front of a computer. In the afternoon, you stay in front of the TV. If you have some exams or tests, you have to stay all afternoon on YouTube looking for videos that explain what you did at school because it's not clear. So, it’s like this all the time.

These difficulties could highlight the need for social support for students in the context of emergency OSL. A participant highlighted, for example, how they found a way to deal with the situation when they could receive greater support from family members:

I was initially at home alone all day because my parents both worked, and I had no connections of any kind. There was only the school that did not know how to organize itself. I found myself overloaded with work because no one thought that it would last for so long. It was very stressful. Then my mom started smart-working, my father started working less, and the school learned to manage this situation, and we are learning how to proceed.

##### **Neutral Changes**

No noteworthy changes were reported when daily routines still revolved around school activities, regardless of whether they were face-to-face or online:

Mornings are more or less similar to the pre-epidemic ones because even if you don’t go to school, in the end, you enter the studio, and you are connected for five hours on a video call, and therefore it is as if you were in school.

### Interpersonal and Communal Factors of Online School Learning

The COVID-19 pandemic and OSL affected social relationships with classmates, teachers, and the school community.

#### Classmates

Analysis of the interviews indicated that several changes occurred in relationships with classmates in terms of a) quality and quantity of relationships among classmates; b) social cohesion, participation, and mutual help; c) awareness related to “in-person relationships”.

##### **Quality and Quantity of Relationships Among Classmates**

Participants reported that in some cases, the interruption of face-to-face interactions and school activities put a strain on peer relations among classmates. Indeed, a tense and nervous climate during OSL could arise: “It’s a very, very tense atmosphere. Just yesterday, we had some problems, and they were lucky that I didn’t kill anyone”. OSL made communication worse, facilitating quarrels and controversies between classmates: “relationships have changed. Before everything was fine. However, now nothing is fine, never”.

Another significant change in quality and quantity of peer relationships concerns the re-evaluation of friendships at school – in both positive and negative ways. The emergency and OSL allowed participants to think about the nature of their relationships and to consider only the positive ones (in terms of authenticity, mutual support, empathy, and shared emotional connection). This meant ending some relations and starting new ones: “I have become a little more friendly with some classmates with whom there was not so much confidence before. But now, I am no longer close to girls I used to talk to”.

##### **Social Cohesion, Participation, and Mutual Help**

On one side, participants reported that participation among their classmates decreased, while absenteeism increased: “We have had this problem in class this year, we are not very united. You could also tell from online lessons where some never showed up, besides the 5-6 people who always participate”. Indeed, OSL did not facilitate peer collaboration and was particularly detrimental when relationships were complex before the COVID-19 pandemic. Instead, students perceived an increased individualism, passivity, and lack of interest for others: “Slowly we began to turn off the cameras, mute the microphones, always moving apart a little more”; “perhaps we are more detached”.

Conversely, there was an opposite effect on previously healthy peer relationships, which had grown stronger. The activation of mutual help and support emerged as critical aspects for participants during the lockdown and helped reinforcing a previously cohesive class as a group: “We were very close even before, but now we are all in the same boat, so we help each other. When someone doesn't understand, we try to help them”. Participants underlined that mutual help regarded learning or lessons and promoted motivation and activation among peers. Mutual help seemed to have strengthened classmates’ relationships and allowed participants to feel less alone during the lockdown, as they could share anxieties, fears, and concerns with their peers: “When I am alone and think about the exam, it scares me much more than with a friend. Things get smaller if I talk to someone else sharing my situation. I feel less alone”.

##### **Awareness Related to “In-Person Relationships”**

Finally, results showed that participants were induced to reflect on their “in-person relationships" at school and re-think the significance of personal contact compared to contact mediated by technology. Participants seemed to be aware of their feelings towards their peers and the importance of sharing face-to-face moments, experiences, and relevant phases of their life (including the end of school or the final exam): “My school friends and I used to talk very often, even about small things, but in this period, we realized that we miss being together and that we love each other more than we thought”.

#### Teachers

##### **Availability**

Participants expressed several needs and expectations that they felt towards teachers during the emergency. In the context of the new experience of OSL, prompt availability of teachers for clarifications and discussions was perceived as an added value to learning:

Teachers were always available. We could always contact them. You have fewer distractions than at school: at school, classmates talk to you or each other, the last rows are always the ones where you pay less attention. Online, you can always ask questions and pay more attention.

##### **Social Support**

Participants appreciated particularly receiving social support beyond the mere teaching and clarification of content. Students appreciated teachers who were understanding, supportive, and “human”:

My teachers are very good. They were always available. They gave us their numbers; when we want to, we call them; even during the quarantine, they told us: ‘If you want to talk to someone, maybe an adult but not your father, call us, no problem’.

Students also appreciated seeing their teachers' efforts and commitment. This helped students feel valued and supported in addressing this experience: “All the teachers got involved, which showed that they cared”.

At the same time, when these capabilities were missing, teachers were perceived negatively by students, who reported feelings of disappointment, frustration, and sorrow: “Very often teachers are poorly altruistic. They don't understand that everything is difficult and give assignments like crazy”; “I’d say almost a lack of humanity”.

#### School Community

The crisis affected adolescents' experience of schools as communities and “places” to live and share everyday life. The analysis identified relevant changes related to the schools’ capacity to respond to students’ emotional, relational and learning needs in pandemic times. The change to OSL could be perceived as depriving students of the essence of the school experience, identified in the value of face-to-face and extra-curricular activities, as well as relationships:

During the lockdown, the school was the thing I miss the most. I am a school representative, and for me, school is a very important thing. I like studying, being active at school, meeting friends. I miss this part so much. Online classes have never replaced what school was for me*.*

The experience of OSL as an everyday environment was characterized negatively. Some students reported that the online school had been a place that rejected them, making them feel inadequate and uncomfortable, emphasizing the association of negative feelings to OSL:

Honestly, my problem is that beyond the teacher’s way of doing, who can try to engage as much as they want, it is the unwelcoming environment that, at least in my case, rejected me. So, I am happy to no longer have to deal with distance learning.

## Discussion

The results of this work offer an insight into the lives of adolescents during the spring 2020 lockdown in Italy, offering the opportunity “to hear their voice” on a situation that has seen them excluded from the major part of the public debate. The study aimed to explore students’ experience of the abrupt transition to emergency online school learning following the spread of the COVID-19 pandemic. To analyze adolescents’ perspectives on how this shift influenced changes in their learning and relationships, we conducted and analyzed interviews with upper secondary school students from Italy, adopting an ecological perspective to understand how structural/organizational, personal/psychological, and interpersonal/communal factors within the school context where implicated in the process. The findings do not allow us to identify the changes that came with adopting OSL as strictly negative or positive, in line with existing evidence highlighting that the first wave of the COVID-19 pandemic was anything but an unequivocal experience to adolescents (Fioretti et al., 2020). OSL has made study and concentration more difficult, but it has also made it possible to experiment with a new personal organization of learning; it has inhibited relationships with peers and teachers, but distance has also allowed adolescents to look at relationships and at their schools with a greater critical sense rather than take them for granted. The complexity of students’ experiences of both challenges and potential strengths of adopting OSL under emergency circumstances was highlighted at different ecological levels of examination. The analysis of students’ perceptions identified some of the crucial factors that need to be taken in consideration for a better development and adaptation of OSL methods under challenging circumstances: organizational preparedness and adaptation of teaching methods; facilitation of self-regulation and time management; provision of social support and collaborative learning.

### Structural and Organizational Factors: The Importance of Preparedness

When referring to structural and organizational dimension of OSL, students revealed a sense of confusion toward the new teaching method and a denunciation of schools’ lack of organization in addressing the crisis. The Italian school has found itself substantially unprepared to manage OSL. Although there has been talk of e-learning for at least twenty years, the “experiment” imposed by the pandemic has revealed that Italian schools did not have the resources to address a transition to OSL adequately. The shortage does not only concern the technical devices that, at least on a general level, are present in many schools, but rather – the regulatory vacuum and the absence of valuable coordinates for organizing remote activities and, above all, the lack of training of pupils and teachers in the use of technologies. The activities were launched under the banner of improvisation, in a context of great confusion, entrusted to the free initiative of school managers and the adaptive capacity of teachers. For most students, however, it was clear that emergency OSL was the very first experience for the school with this method. In general, they seemed to think of the experience in terms of a temporary solution – the only possible one – to a pressing problem.

In line with previous evidence (Yates et al., 2020; Niemi & Kousa, 2020; Pınar & Dönel Akgül, 2020), our interviewees recognized the flexibility and accessibility that OSL offered. Adopting creative and flexible technological solutions in teaching and providing greater choice for students (e.g., multiple online tools, planned examinations) were seen as more motivating factors and could reduce the pressure related to OSL. The acquirement of new competencies might be perceived as a positive side effect of emergency OSL. For example, the interviews suggest an increased awareness of the possibility of learning through the use of technology among students. In the future, it would be interesting to understand how this ongoing process of discovery of the educational utility related to digital technology can be integrated into post-emergency teaching.

### Personal and Psychological Factors: Nourishing Autonomy

For some students, a very appreciated benefit of OSL during this period was the increase of self-regulation. The possibility of having an active role in the management of one’s learning process was a novel experience for students that could promote better outcomes and greater satisfaction. Attempting to organize studying through asynchronous learning was perceived positively by participants and was framed as allowing their growth, also in terms of preparing them for university and more autonomous learning methods. As Bruining et al. (2020) noted, the crisis, home confinement, and remote learning offered opportunities for adolescents who often struggle in a traditional school environment to succeed at an individualized pace of learning. However, previous research has suggested that such a perspective could be limited to students who exhibit greater self-direction and efficacy (Blanco et al., 2020; Iivari et al., 2020; Procentese et al., 2020).

The shift to OSL has allowed the questioning of pre-pandemic routines and has promoted a reflection about the use of time (the pace of daily life), as well as the development of hobbies and talents. Home-based lectures offered youths more time to discover new passions, providing a greater sense of control on and meaning to their lives. The students seemed to suggest an idea of a school allowing them to learn while being truly capable of encouraging everyone's talents.

However, in line with recent literature (Sutarto et al., 2020), our results also show that students felt overloaded by emergency OSL. A recent study highlighted the phenomenon of *school burnout*, referring to the stress reaction of teachers engaged in OSL: faced with new demands and instructional requirements, teachers show high levels of stress and anxieties due to the current state of education and the pandemic (Pressley, 2021). Our results suggest that a similar stress-based response can be found in students, as they perceive school demands, amount of required study, and loneliness linked to the emergency OSL as overwhelming. Further research should investigate the importance of perceived school demands as to students’ well-being, as well as potential strategies for the prevention of stress-based responses.

### Interpersonal and Communal Factors: Social Support and Collaboration

Despite benefits of personalized and self-regulated learning, an individualized approach could be inadequate for some students or circumstances. Indeed, students’ accounts of the emergency schooling stressed the need for social support, mutual help, and collaboration with teachers and classmates. This suggests that cooperative learning should not be overlooked but integrated with independent activities. Indeed, collaborative activities and teamwork were absent from students’ accounts, suggesting that schools and teachers were generally not prepared to offer a structured approach to facilitating cooperative learning during OSL. The issue, however, can be related to the existing difficulties of Italian schools in promoting student cooperation (OECD, 2020). It would be necessary for teachers to receive adequate training on how to balance providing autonomy, support, and cooperation in applying OSL methods, even if in emergency settings.

Our findings show a different evaluation of the online school as an institutional organization and community: if the first receive decidedly negative evaluations, the second shows both challenges and strengths. The centrality of relationships with teachers and classmates was evident in this sense. Among the potential strengths of OSL, teachers could modulate their teaching skills concerning the situation and the students they faced. Adaptation and change of teacher-student interactions were essential in successfully addressing the new and unknown way of schooling. Diverging from the traditional role and teaching space enabled the student-teacher relationship to survive the shift from face-to-face to online classes and allowed students to re-evaluate the intergenerational relationships with their teachers. When this was not possible, the school failed to sustain students’ capacity to develop autonomy in the learning process within a relational context. When students perceived a lack of support and adaptation of the teacher-student relationship, they expressed a profound disappointment and a sense of abandonment. Indeed, participants were more prone to reproach teachers for not caring and not sufficiently considering their needs rather than for having difficulties in using technologies. The emphasis put on these aspects testifies to the importance that adolescents attribute to the school and their teachers in their education, personal development, and well-being.

Secondly, in some cases, the students found a context of solidarity and social support in the class, which represented an important anchor for coping with the situation (Gatti & Procentese, 2021; Procentese et al., 2020). The results suggest, in support of the work of Peterson et al. (2020), that pre-existing strong classmate relationships helped students to cope better, even in unfavorable school contexts, and to find stronger resilience than before. Conversely, weak relationships were compromised by OSL, making social networks pruning happen earlier in life (adolescence vs. adulthood; Wrzus et al., 2013). It would be important to help young people who have experienced situations of stress and conflict to manage these situations in a constructive way and strengthen the supportive capacity of their relational environments.

### Limitations

This study has some limitations, including its explorative nature and the specific period in which the data were collected. The interviews were conducted at the tail of the first COVID-19 wave and the duration of restrictions for education was uncertain. Further research, with longitudinal designs, could unveil the impact of the lengthening of online-based schooling on students. Moreover, our study did not include information on possible conditions of disadvantage among students, nor did we recruit participants from diverse migrant or ethnic background. Considering the persisting digital gap in education (e.g., Hall et al., 2020; Asanov et al., 2021), future research should provide greater understanding of adolescents’ experiences of OSL in interaction with their socio-economic and cultural background.

## Conclusions

The pandemic has highlighted weaknesses of the Italian school system and the importance of introducing changes in its mode of operation, but it has also revealed the need to reflect on its training and educational purposes. Exploring students’ experiences of the emergency adoption of online schooling during the first lockdown in Italy with an ecological approach, the study highlights adolescents’ perspectives on the aspects of learning environments valued for their well-being and supportive of coping with critical situations. These include organizational flexibility, creative and innovative teaching approaches, the opportunity to be autonomous and proactive in learning, supportive relationships with teachers and classmates, suggesting the need to adopt multilevel approaches not only to the analysis but also to the planning of interventions in schools.

## Data Availability

The data that support the findings of this study are available from the corresponding author upon reasonable request.
